# The effectiveness of exercise-based interventions on muscle mass, muscle strength, functional performance, aerobic capacity, and health-related quality of life in adults with malignant lymphoma undergoing chemotherapy: a systematic review of randomized controlled trials

**DOI:** 10.2340/1651-226X.2025.42056

**Published:** 2025-01-28

**Authors:** Charlotte Grønset, Magnus N. Bech, Mary Jarden, Nina Høgdal, Martin Hutchings, Charlotte Suetta, Jan Christensen

**Affiliations:** a Department of Occupational Therapy and Physiotherapy, Copenhagen University Hospital Rigshospitalet, Copenhagen, Denmark; b Department of Hematology, Copenhagen University Hospital Rigshospitalet, Copenhagen, Denmark; c Department of Clinical Medicine, Faculty of Health, University of Copenhagen, Copenhagen, Denmark; d Department of Geriatric and Palliative Medicine, Copenhagen University Hospital – Bispebjerg and Frederiksberg, Copenhagen, Denmark

**Keywords:** Lymphomas, hematology, chemotherapy, exercise, physical activity

## Abstract

**Purpose:**

This study aims to identify and summarize evidence on the effectiveness of exercise-based interventions on muscle mass, muscle strength, functional performance, aerobic capacity, health-related quality of life (HRQoL), feasibility of the interventions, in patients with malignant lymphoma undergoing chemotherapy.

**Methods:**

A systematic search was conducted in six electronic databases and trials registers on November 15, 2023. Peer-reviewed randomized controlled trials (RCTs) comparing exercise intervention with controls/usual care in adults (≥18 years) diagnosed with Hodgkin’s lymphoma and non-Hodgkin’s lymphoma undergoing chemotherapy were considered for inclusion. All study authors were contacted to obtain unpublished subgroup data. Two reviewers independently screened and extracted data and assessed the quality of evidence using the revised Cochrane risk-of-bias tool for randomized trials.

**Results:**

Six RCTs published between 2009 and 2021, with 838 participants, were included. Due to clinical heterogeneity, a meta-analysis was not feasible, therefore the results were synthesized narratively. Exercise interventions during treatment were found to be feasible with few adverse events reported. The included studies indicate positive effects of exercise during chemotherapy on muscle mass, muscle strength, functional performance, aerobic capacity, and HRQoL compared to usual care.

**Interpretation:**

Despite extensive search criteria, a limited number of heterogenous studies were eligible, which may explain the very low certainty of evidence for all outcomes. Nonetheless, exercise-based interventions conducted during treatment were feasible, safe and potentially effective. Further studies are needed to guide future exercise recommendations for these patients.

## Introduction

Lymphomas are cancers of the lymphatic system. They are categorized into Hodgkin’s lymphoma (HL) and non-Hodgkin’s lymphoma (NHL), with diffuse large B-cell lymphoma (DLBCL) being the most common NHL [1]. In 2020, the global incidence of NHL and HL was estimated to 628,000 [2]. The incidence of malignant lymphoma is increasing primarily due to the aging population [3]. Early detection and advances in treatment [4] have contributed to improved survival rates, leading to an increase in the prevalence of lymphoma survivors. The 5-year relative survival rates are 75% for NHL and 90% for HL according to US data [4]. Patients with malignant lymphomas undergo intense treatments, including prolonged chemotherapy, which can lead to complications such as infections, fatigue, malnutrition, and muscle dysfunction (loss of strength, mass, and functional performance) [5]. These complications can lead to hospital admission, worsening side effects, decreased health-related quality of life (HRQoL), and may impact survival [6–8]. Strong observational evidence supports the impact of muscle dysfunction [9] on cancer treatment and outcomes, such as chemotherapy mediated toxicity, resulting in reduction of chemotherapy doses and finally treatment interruption [10]. Accordingly, cancer patients are advised to maintain an active lifestyle and adhere to public health guidelines for physical activity during treatment [11, 12]. Exercising during cancer treatment is gaining momentum, supported by numerous studies showing positive physiological and psychosocial effects to mitigate symptoms and side effects [13]. A previous overview of systematic reviews [14] found exercise beneficial for fatigue, psychological symptoms, and quality of life (QoL) in lymphoma patients, but the evidence was limited and highly heterogeneous. In 2022, Aljohi et al. [15] highlighted large variation in patient populations and interventions, limiting the generalizability of the findings. This emphasizes the need for further review of the effectiveness of exercise in lymphoma patients during treatment. To our knowledge, this is the first systematic review focused solely on exercise during initial treatment and its effectiveness on muscle mass, strength, and functional capacity and performance in lymphoma patients.

### Objectives

This review aims to assess the effectiveness of exercise intervention on muscle mass, strength, functional performance, aerobic capacity, and HRQoL in adults with malignant lymphoma undergoing chemotherapy. In addition, it will evaluate feasibility, adverse events, and the impact of type, intensity, duration, and delivery mode on outcomes. Furthermore, this study will examine feasibility, adverse events, and the impact of type, intensity, duration, and delivery mode on outcomes.

## Methods

### Registrations

This systematic review was conducted according to the recommendations of the Cochrane Collaboration [16] and reported according to the Preferred Reporting Items for Systematic reviews and Meta-Analyses (PRISMA) and Synthesis without meta-analysis (SWIM) guidelines [17, 18]. The study protocol was registered in the International Prospective Register of Systematic Reviews (PROSPERO) database in May 2022 (registration number CRD42022336588).

### Eligibility criteria

Randomized controlled trials (RCT) investigating the effectiveness of an exercise intervention on at least one of the following outcomes: muscle mass, muscle strength, functional performance, aerobic capacity, and HRQoL were considered eligible if participants were ≥18 years, diagnosed with NHL or HL, and undergoing chemotherapy. Studies with mixed samples, including malignant lymphoma patients, were eligible for sub-group analysis. Exercise-interventions had to focus on resistance training, aerobic training, or a combination of these. Multifaceted interventions were included if the exercise intervention was an essential part of the intervention. Delivery mode could be supervised or unsupervised, individual, or group-based, and conducted at a center or at home. Studies on low-intensity interventions such as yoga, tai chi, and qigong were excluded. Control interventions were defined by the study, such as no exercise or usual care.

### Information sources and search strategy

A systematic search was conducted on November 15, 2023, in MEDLINE (ovid), EMBASE, CINAHL, the Cochrane Central Register of Controlled trials (CENTRAL). Additionally, the WHO International Clinical Trials Registry Platform (ICTRP) and ClinicalTrials.gov and were screened to identify ongoing trials. The search strategy was developed with a research librarian from the Medical Library, Copenhagen University Hospital. The search matrix consisted of relevant keywords and MeSH/Thesaurus terms for (1) malignant lymphoma and (2) exercise. An RCT-filter recommended by the Cochrane Handbook [16] was used in Medline. The search filter was adjusted with a wild card in radomised for improved sensitivity. No language or publication date restrictions were applied. Supplementary Appendix A presents the search strings for all databases and trials registers.

### Selection process

Search results were imported into Covidence (Veritas Health Innovation, Melbourne, Australia, available at www.covidence.org). Two authors (CG and MNB) independently screened all titles, abstracts and then all articles in full text. Disagreements were resolved with a third reviewer (JC). The first author and corresponding authors were contacted by email for clarification on study information and diagnosis-specific data.

### Data collection process

CG and MNB independently extracted data; disagreements were resolved by consulting JC. Microsoft excel spreadsheet was used for data extraction of all a priori defined variables: author, publication year, country, age, body mass index (BMI), sex proportion, exclusion criteria, type of diagnose, number of estimated participants, outcome measure with calculated power, number of eligible participants, number of participants randomized to intervention or randomized to control group, frequency of exercise per week, intensity of exercise, type of intervention, supervised or non-supervised intervention, duration of exercise per session, delivery mode, time of intervention related to treatment, length of intervention, setting, control group intervention, time of assessment, assessment method, type of outcome, estimates, variance, *P*-values for the effect on relevant outcomes, and adverse events.

### Data items

#### Timepoint of interest

The primary time point of interest for all outcomes was end of exercise intervention.

#### Primary outcomes

##### Muscle mass

Terms such as lean mass, lean tissue mass, fat-free mass, and muscle mass are commonly used as approximations to describe muscle mass. Measures include computed tomography (CT), magnetic resonance imaging (MRI), dual energy X-ray absorptiometry (DEXA), or bioelectrical impedance spectroscopy (BIS).

#### Muscle strength

Measures of muscle strength refer to the ability of muscles to produce resistance or movement against external forces during contraction. This might be one repetition maximum (1RM) or other proxies of augmented muscle contraction.

##### Functional capacity and performance

Functional capacity and performance are constructs used to understand physical functioning. Functional capacity refers to physical abilities assessed under standardized conditions such as gait speed test, 6-min walking distance test, Timed Up and Go test and repeated chair stand test. Functional performance reflects an individual engagement in physical activities, often derived from self-reported questionnaires.

#### Secondary outcomes

##### Aerobic capacity

Measures of aerobic capacity include any objective measure of the ability of heart and lungs to deliver muscle oxygen consumption, such as maximal or peak oxygen consumption. Changes could be an increase in peak oxygen consumption obtained from a maximal cardiopulmonary exercise test, or as a decrease in submaximal oxygen uptake at a given workload, or a decrease in submaximal heart rate at a given workload.

##### Health-related quality of life

Measures of HRQoL outcomes include both generic and disease-specific patient-reported outcome measures.

#### Additional outcomes

Feasibility outcomes (e.g. retention, drop-outs, and adherence) and safety assessed as the number of adverse events.

### Study risk of bias assessment

The Cochrane Collaboration’s Risk of Bias tool version 2 [19] was used to assess risk of bias (RoB) in the included studies. CG and MNB independently evaluated each of the five domains: bias from randomization process, deviation from intended interventions, missing outcome data, outcome measurement, and selection of reported results. The RoB was categorized as high, some concerns, or low.

### Synthesis considerations

The study was designed as a systematic review with the potential for a meta-analysis (PROSPERO registration number CRD42022336588). Hence, due to clinical heterogeneity a narrative approach was used to summarize the data.

For studies reporting data on a sample including other diagnoses than lymphoma, the authors will be contacted to request data specifically for the lymphoma subset. If additional data are provided, appropriate data management and statistical analysis of between group differences will be conducted. Results for the outcome of interest will be primarily presented based on the reported aggregate data in the original articles, as mean with 95% confidence intervals (95% CI) or medians with interquartile range (IQR). Missing values for 95% CI will be calculated from the presented data when possible, using the method described by Bland and Altman [20]. Weighted means of patient’s characteristics will be calculated and presented when appropriate. *P*-value <0.05 will be considered statistically significant.

### Reporting bias assessment

To assess outcome reporting bias, protocols and trial registries such as ClinicalTrials.gov and WHO databases will be searched to compare intended and analyzed outcomes.

### Certainty assessment

If a meta-analysis is performed, the assessment of the body of evidence for the effect of exercise-based interventions will be conducted using the Grading of Recommendations Assessment, Development and Evaluation (GRADE) tool. If a narrative analysis is performed, GRADE assessment will not be conducted.

## Results

### Study selection

**Figure 1:**
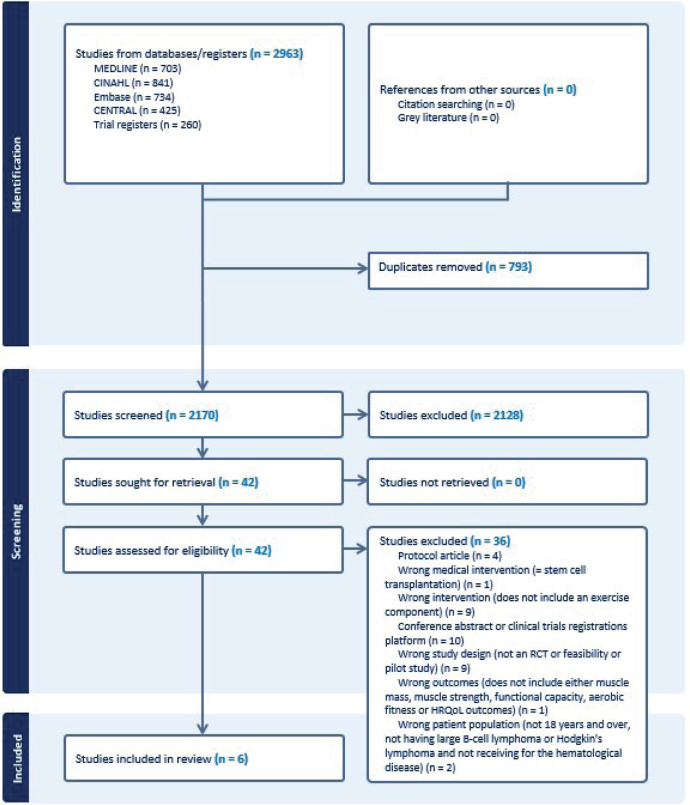
Flowchart of the study selection process.

The search yielded 2,963 hits. After removing 793 duplicates, 2,170 unique studies were screened. Figure 1 shows the selection process and exclusion reasons. Finally, six studies with 838 participants were included.

### Study characteristics

In total, six RCTs were included for analysis [21–26]. Two studies included patients with mixed cancer diagnosis [21, 22] with 3.0% and 8.2% of the patients included having a lymphoma diagnose, respectively. Three studies included patients with various hematological malignancies of which 18.8%, 26% and 78.5% of the study populations were diagnosed with malignant lymphoma, respectively [24–26]. One study solely included patients with malignant lymphoma [23]. From one study [26], relevant sub-sample data of nine patients ≥18 years, with malignant lymphoma could be obtained. Data from this sub-sample were analyzed using a paired *t*-test and included. Across the six studies, patients received various medical treatments, as exemplified by one study reporting 59 different chemotherapy regimens during the study period [21]. Table 1 presents an overview of the included study characteristics. Sample sizes ranged from 43 to 301 participants, with a weighted mean age of 57.5 years.

**Table 1:** Study designs.

Author, year, country	Participants’ characteristics	Exclusion Criteria	Intervention				Control group	Timing of tests	Extracted outcomes
			Delivery	Type	Frequency and duration	Intensity			
**Adamsen** et al., 2009, [21] Denmark	**Diagnoses**: Various cancers, Hodgkin’s lymphoma, non-Hodgkin Lymphomas, acute leukemia, chronic leukemia **Disease stage**: NR **Participants (*n*)**: 269 IG:135 CG: 134 **Sex:** F:196 (73%) M:73 (27%) **Mean age:** 47 years **Performance status:** By WHO PS 0–1: 225 (84%) PS >2: 44 (16%) **Treatment**: 59 different chemotherapy regimens	Brain or bone metastases, thrombo-cytopenia, myocardial infarction within past 3 months, uncontrolled hypertension	Hospital-based, supervised	Aerobic, strength, relaxation, body awareness Massage	6 weeks multimodal exercise program 15 min aerobic 3x/week 45 min RT 3x/week 30 min relaxation 4x/week 90 min body awareness 1x/week 30 min massage 2x/week	Strength: 5–8 rep. at 70% – 100% of 1RM Aerobic: 70–250W workload and 85% – 95% max HR Low intensity interventions: Body awareness, relaxation, and massage sessions	Usual care. Allowed to freely increase physical activity	Baseline 6 weeks	Aerobic capacity Muscle strength Functional performance HRQoL
**Arrieta** et al., 2019, [22] France	**Diagnoses**: Various cancers, diffused large B-cell lymphomas, T-peripheral, lymphocytic, lymphoplasmacytic, follicular, mantle, marginal zone **Disease stage:** NR **Participants, (*n*):** 301 (300 analyzed) IG: 150 CG: 151 **Sex**: F: 180 (60%) M: 120 (40%) **Mean age**: 77 years **Performance status:** BY ECOG PS 0: 160 (53%) PS 1: 89 (30%) PS 2: 12 (4%) NR: 39 (13%) **Treatment:** Surgery, chemotherapy, Radiation, Hormone therapy, Targeted therapy	ECOG PS >2, serious psychiatric or cognitive problems. inability to walk, palliative care	Home-based, unsupervised	Strength, balance, proprioception, flexibility, and aerobic training.	One-year multimodal exercise program conducted 2x/week. Phone calls twice a month during the first 6 months and then monthly until 1 year with exercise instruction including progression and regression of the exercises based on feedback and for motivational purpose.	Individual according to patients’ capability and motivation	Usual care and a booklet with recommendations for physical activity	Baseline 3 months 6 months 12 months 18 months 24 months	Functional performance
**Courneya** et al., 2009, [23] Canada	**Diagnoses:** NHL indolent, NHL, aggressive, HL **Disease stage for patients with evidence of disease:** Stage 1: 20.9%, Stage 2: 26.7%, Stage 3: 19.8%, Stage 4: 32.6% **Participants, (*n*) =** 122 IC: 60 CG:62 **Sex**: F: 50 (41%) M: 72 (59%) **Mean age**: 53 years **Performance status:** NR **Treatment:** 55.7% off treatment, 44.3% chemotherapy	Uncontrolled hypertension, cardiac illness	Hospital-based, Supervised	Aerobic training	12 weeks exercise program 45 min aerobic training 3x/week	60% of peak power output, increased to 75% by the fourth week	Usual care. The control group were asked not to increase exercise above baseline level.	Baseline 12 weeks 6 months	Body composition Aerobic capacity Functional performance HRQoL
**Oechsle** et al. 2014, [24] Germany	**Diagnoses:** Acute myeloid leukemia, non-Hodgkin’s lymphoma, multiple myeloma, relapsed germ cell tumor **Disease stage**: NR **Participants, (*n*):** 58 (48 analyzed) IG:29 CG:29 **Sex:** F: 50 (41%) M: 72 (59%) **Mean age:** 52 years **Performance status:** By Karnofsky IG: 92 CG: 89 **Treatment:** Chemotherapy, high dose chemotherapy with stem cell transplantation	Cardiovascular diseases, tumor infiltration of the skeletal system, epilepsies, rheumatologic diseases, BMI < 18), BMI > 30, cognitive impairment	Hospital-based, supervised	Aerobic and strength training	9–21 days (range 16–33) multimodal exercise program during hospitalization 20 min aerobic training 5x/week 20 min strength training 5x/week	Aerobic: individually tailored. Resistance training: 3 exercises 2 set of 15–26 rep. at 40% – 60% of 1RM	Standard physiotherapy (Including breathing therapy or exercise practices)	Baseline Post-test 12 months	Muscle strength HRQoL
**Streck-mann** et al., 2014, [25] Germany	**Diagnoses:** Hodgkin’s disease, B-NHL, T-NHL, Multiple myeloma **Disease stage:** by Ann Arbor Stage 1: 12.8%, Stage 2: 25.6%, Stage 3: 17.9%, Stage 4: 43.6% **Participants, (*n*):** 56 IG:28 CG:28 **Sex:** F:14 (25%) M:42 (75%) **Mean age:** 46 years **Performance status:** NR **Treatment:** Radiation, Immunotherapy, Chemotherapy, Stem cell transplantation	Bone metastases, severe acute infections, severe cardiac-pulmonary disease, restrictions for physical activity	Hospital-based, supervised	Aerobic and strength training, sensorimotor exercises	36 weeks multimodal exercise program 10–30 min aerobic training 2x/week NR min sensorimotor training 2x/week NR min strength training 2x/week	Aerobic: 60% – 80% of max heart rate Strength training:1 min of maximum force for each of the four exercises. Substituted with a Thera-BandTM if patients were hospitalized. Sensorimotor training: 3 sets of 3 rep. Each rep performed for 20 s.	Physiotherapy	Baseline 12 weeks, 24 weeks, 36 weeks	Functional performance HRQoL
**Munsie** et al., 2021, [26] Australia	**Diagnoses:** Hodgkin’s lymphoma, sarcoma, CNS tumor, germ cell tumor, leukemia, melanoma, Burkitt lymphoma **Disease stage:** NR **Participants, (*n*):** 43 IG:21 CG:22 **Sex**: F: 16 (37%) M: 27 (63%) **Mean age:** 21 years **Performance status:** NR **Treatment:** Chemotherapy or combined chemotherapy and radiation	Surgery only, insufficient English competency or cognitive impairment, medically unable to participate, pregnant or lactating, or had a life expectancy <6 months	Hospital-based, supervised	Aerobic, strength, and flexibility exercises	10 weeks in all, 60 min multimodal exercise program Up to 30 min aerobic training 2x/week NR min strength training 2x/week	Aerobic: 60% – 85% of maximal HR Strength training: 60% – 80% of 1RM for up to eight exercises. Body weight, dumbbell, and machine-based exercises were used. NR for set and rep.	Standard care with general physical activity advice. And weekly contact for monitoring any treatment-related toxicities.	Baseline 10 weeks	Body composition Aerobic capacity Muscle strength Functional performance HRQoL

BMI: Body Mass Index; B-NHL: B-cell non-Hodgkin lymphoma; CG: Control group; ECOG: The Eastern Cooperative Oncology Group; F: Female; HR: Heart rate; HRQoL: Health Related Quality of Life; IG: Intervention group; M: Male; Min: Minutes; N: Number; NR: Not reported; PS: Performance status; Rep: Repetition; RM: Repetition maximum; S: second; T-NHL: T-cell non-Hodgkin lymphoma; WHO: World Health Organization.

### Exercise-based interventions

The exercise-based interventions varied by type, intensity, duration, and mode of delivery. A summary is outlined in Table 1.

### Risk of bias in studies

**Figure 2:**
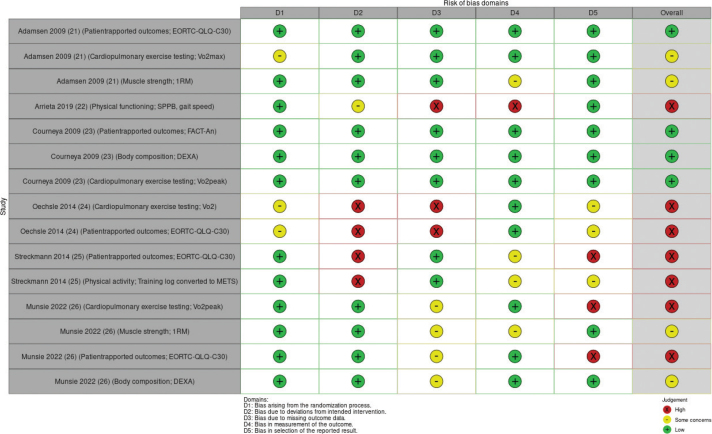
‘Traffic light’ plots of the domain-level judgments for each individual result.

**Figure 3:**
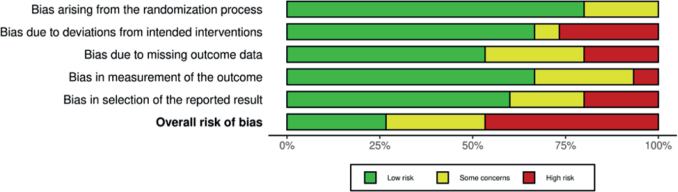
Weighted bar plots of the distribution of risk-of-bias judgments within each bias domain.

The six trials demonstrated varying levels of RoB across the domains. Four RCTs had an overall high RoB [22, 24–26], one had some concerns [21], and one had low RoB [23]. The RoB in individual studies is summarized in an evidence synthesis in Figure 2. Weighted bar plots of the distribution of RoB judgments within each bias domain in Figure 3. Authors’ judgments are further described in Supplementary Appendix B.

### Effectiveness of exercised-based interventions

The effectiveness of exercise-based intervention reported in the studies is presented in Table 2.

**Table 2:** Study results from baseline to postintervention; body composition, physical and QoL outcomes.

Author year	Muscle mass		Muscle strength		Functional performance		Aerobic capacity		QoL	
	Outcome measure	Results	Outcome measure	Results	Outcome measure	Results	Outcome measure	Results	Outcome measure	Results
**Adamsen** et al. [21], 2009	-	-	1RM tests Leg-press, Chest-press and Pull-down, mean (SD)	Leg press IG: Change from 100.8 (30.5) to 132.4 (42.3) CG: change from 107.6 (33.3) to 110.4 (36.0) *P* < 0.0001 Chest press IG: change from 37.9 (15.6) to 45.2 (17.9) CG: change from 107.6 (33.3) to 110.4 (36.0) *P* < 0.0001 Pull-down IG: change from 39.6 (14.0) to 47.2 (14.4) IG: change from 42.0 (16.3) to 42.8 (16.1) *P* < 0.0001	SF 36 physical functioning score (*p*) mean (SD)	IG: change from 84.3 (13.7) to 88.2 (13.2) CG: change from 83.6 (14.8) to 84.3 (16.2) *P* = 0.01	VO_2_ max test (l/min), mean (SD)	IG: change from 1.82 (0.4) to 1.96 (0.5) CG: change from 1.90 (0.5) to 1.88 (0.5) *P* < 0.0001	EORTC-QLQ-C30 score (*p*), mean (SD)	IG: change from 63.8 (21.1) to 67.2 (20.3) CG: 60.2 (22.4) to 63.3 (22.4) *P* = 0.4
**Arrieta** et al. [22], 2019	-	-	-	-	SPPB score (*p*), Median (IQR)	IG: change from 10 (8–11) to 10 (7.5–12) CG: change from 10 (8–11) to 10 (9–11) *P* = 0.999	-	-	-	-
	-	-	-	-	Gait speed (m/s), median	IG: change from 0.78 to 0.91 CG: change from 0.80 to 0.92 *P* = 0.171	-	-	-	-
**Courneya** et al. [23], 2009	Lean body mass by DEXA scans (kg), mean (SD)	IG: change from 52.4 (10.9) to 52.3 (11.5) CG: change from 49.9 (10.0) to 49.8 (9.9) *P* = 0.008	-	-	TOI-An, physical functioning score mean (SD)	IG: change from 100.1 (21.9) to 109.3 (18.7) CG: change from 105.1 (21.1) to 105.4 (22.0) *P* = 0.012	VO_2_ peak (l/min), mean (SD)	IG: change from 2.02 (0.66) to 29.4 (8.6) CG: change from 1.98 (0.71) to 1.96 (0.69) *P* < 0.001	FACT-An score (*p*) mean (SD)	IG: change from 140.7 (26.8) to 151.4 (21.7) CG: change from 147.1 (24.3) to 148 (25.7) *P =* 0.021
**Oechsle** et al. [24], 2014	-	-	Estimated 1 RM, mean (SD)	*Only reported for the IG* Bridging IG: change from 48.5 (24.7) to 57.6 (33.7)*P*-value NR Sit-ups IG: change from 35.8 (15.2) to 41.8 (34.4) *P-*value NR Theraband exercise IG: change from 41.5 (24.1) to 56.3 (43.6) *P* = 0.04	-	-	-	-	EORTC-QLQ-C30 (end -points)	50 vs. 50 *P* = 0.66
**Streck-mann** et al. [25], 2014	-	-	-	-	Metabolic Equivalent of Task (MET)	IG: 2.5 (–57 to 33) CG: 0 (–27 to 30)	-	-	EORTC-QLQ-C30 score (*p*)	IG: 12 (–67 to 67) CG: –1 (–66 to 42) *P =* 0.113
**Munsie** et al. [26], 2021	Lean body mass by DEXA- scans (kg), mean (SD)	IG: changed from 52.1 (6.8) to 52.5 (5.6) CG: changed from 46.4 (10.9) to 44.9 (12.4)	1RM tests Leg press, Chest press and Seated row, mean (SD) Grip strength by dynamo-meter, (kg) mean (SD)	Leg press IG: Change from 98.0 (37.0) to 115.5 (42.2) CG: change from 78.6 (20.2) to 73.1 (24.8) Chest press IG: change from 37.0 (15.1) to 39.5 (13.3) CG: change from 26.4 (12.6) to 27.6 (11.9) Seated row IG: change from 54.0 (18.5) to 55.0 (13.7) CG: change from 43.8 (10.3) to 43.8 (13.8) Handgrip IG: change from 42.5 (9.6) to 40.4 (9.5) CG: change from 33.7 (16.5) to 34.2 (19.3)	5 repeated STS test mean (SD)	IG: change from 9.4 (2.0) to 9.3 (2.6) CG: change from 11.8 (5.2) to 11.9 (5.2)	Predicted VO_2_ max mean (SD)	IG: change from 32.1 (5.5) to 32.6 (6.1) CG: change from 28.1 (3.2) to 24.6 (4.8)	EORCT-QLQ-C30 score (*p*) mean (SD)	IG: change from 68.4 (16.2) to 73.6 (20.7) CG: change from 72.0 (19.1) to 68.8 (22.0)

CG: control group; DEXA: Dual-Energy X-ray Absorptiometry; EORTC QLQ-C30: the EORTC Core Quality of Life questionnaire. Scores range from 0–100. Higher scores indicate better QoL, FACT: An Functional Assessment of Cancer Therapy-Anemia, scores range from 0 (low) to 188 (high). IG: intervention group; IQR: interquartile range; kg: kilograms; l/min: liters per minute; m/s: meters per second; p: points; QoL: Quality of life; RM: repetition maximum; SD: standard deviation; SF 36: The 36-Item Short Form Health Survey, physical functioning scores range from 0 to 100. A high score represents a high level of functioning, STS: sit to stand; SPPB: Short Physical Performance Battery – The scores range from 0 (worst performance) to 12 (best performance), TOI – An The Trial Outcome Index Anemia scores range from 0 to 136. Higher scores reflect better QoL, VO_2_ oxygen consumption.

### Synthesis of the results on feasibility and adverse events

All included studies presented a power calculation. One was underpowered due to low recruitment [25]. The weighted mean recruitment rate reported by the five included studies [21–23, 25, 26] was 46%. Five studies [21–23, 25, 26] reported exercise adherences, measured as the number of attempted trainings sessions, with adherence rates ranging from 65% to 78%. One study [27] reported compliance with the prescribed exercise intensity as 90.7%. The most reported reasons for dropping out were disease progression, treatment-related side effects or lost motivation [23, 24, 26]. Two studies [21, 23] reported adverse events related to the exercise interventions. One study [21] reported an incident but it was not related to lymphoma patients. Another study [23] reported three adverse events related to joint pain. One of the three participants withdrew from exercise, the other two participants continued with a modified exercise program. Results are shown in Table 3.

**Table 3:** Feasibility.

Author, Year	No. of patients eligible for the study	Included patients. (% of eligible)	No. of patients completed post-test (%)	Statistical power calculation. Estimated sample size (included/planned)	Exerciseadherence	Adverse events
**Adamsen** et al., [21] 2009	953	269 (28%)	235 (87%)	Power calculation presented (269/270)	Adherence rate of 71%	No specific adverse event, related to lymphoma patients
**Arrieta** et al., [22] 2019	452	301 (67%)	248 (83%)	Power calculation presented (301/300)	Completed phone calls 81.1% Performed physical activity 70.1%	NR
**Courneya** et al., [23] 2009	474	122 (26%)	117 (96%)	Power calculation presented (122/120)	Attended exercise sessions: 78%	Three adverse events related to exercise
**Oechsle** et al., [24] 2012	NR	58 (NR)	48 (83%)	Power calculation presented (58/48)	NR	No adverse events
**Streckmann** et al., [25] 2013	186	61 (33%)	51 (84%)	Power calculation presented (61/184)	Compliance to all interventions: 65%	No adverse events
**Munsie** et al., [26] 2021	127	73 (57%)	39 (91%)	Power calculation presented (43/36)	Completed the exercise program: 68%	No adverse events

No: number; NR: not reported.

### Synthesis of the results on muscle mass

Two studies evaluated the effectiveness of an exercise-based intervention on muscle mass using DEXA scans, but with contrary results. One study [26] (overall high RoB) showed no significant between-group difference after 10 weeks of multimodal exercise (MD –1.9; 95%CI, –5.0 to 1.3, *P* = 0.2105). In contrast, another study [23] (overall low RoB) found a significant between-group difference in lean body mass after 12 weeks of an aerobic exercise (MD 0.8; 95%CI, 0.2 to 1.4, *P* = 0.008) in favor of supervised aerobic exercise.

### Synthesis of the results on muscle strength

Two studies evaluated the effectiveness of an exercise-based intervention on muscle strength by estimating the 1RM with conflicting results. One study [21] (overall some concern RoB) evaluated three different exercises; chest press, leg press and pull down, and found significant between-group differences after 6 weeks of exercise for all three outcomes: leg press (MD, 29.7; 95%CI, 23.4 to 34.9, *P* < 0.0001), chest press (MD, 7.5; 95%CI, 5.6 to 9.4, *P* < 0.0001) and pull-down (MD, 6.4; 95%CI, 4.5 to 8.3, *P* < 0.0001) in favor of a supervised multimodal exercise-based intervention. In contrast, another study [26] (overall high RoB) evaluated three different exercises; leg press, chest press and seated row and found no significant between-group difference after 10 weeks of exercise: leg press (MD, –23.1; 95%CI, –67.7 to 21.5, *P* = 0.2597), chest press (MD, –1.3; 95%CI, –8.2 to 5.6, *P* = 0.6686) and seated row (MD, –1.0; 95%CI, –13.3 to 11.3, *P* = 0.8529). Moreover, one study [26] (overall high RoB) evaluated the effectiveness of an exercise-based intervention on grip strength assessed with a dynamometer, showing no significant between-group difference (MD, 2.7; 95%CI, –3.9 to 9.2, *P* = 0.3686). Finally, two studies [22, 24] (overall high RoB) evaluated muscle strength, but no between-group results on muscle strength parameters are reported in the articles.

### Synthesis of the results on functional capacity and performance

All six studies evaluated the effectiveness of an exercise-based intervention on functional capacity and performance using different measurements. Two studies used patient reported outcomes (PROs). One study [23] (overall low RoB) used the subscale TOI-An from the FACT-An questionnaire and found a significant between-group difference after 12 weeks of exercise (MD, 9.0; 95%CI, 2.0 to 16.0, *P* < 0.012) in favor of supervised aerobic exercise. Another study [21] (overall some concern RoB) used the MOS SF 36 physical functioning subscale and found a significant between-group difference after 6 weeks of exercising (MD, 4.4; 95%CI, 1.1 to 7.7, *P* = 0.01). Two studies used objective measures. One study [22] (overall high RoB) used The Short Physical Performance Battery (SPPB) [28] and found no significant between-group difference (*P* = 0.772) after 1 year of exercise. Another study [26] (overall high RoB) used the Five Times Sit to Stand Test and found no significant between-group difference after 10 weeks of exercise (MD, 0.3; 95%CI, –2.6 to 3.1, *P* = 0.8417). Finally, one study [25] (overall high RoB) evaluated the effectiveness of an exercise intervention on functional performance with estimation of metabolic equivalent (MET) using a training logbook and found a significant between-group difference after 36-weeks of exercise (MD, 1.08; 95%CI, 0.3 to 4.7, *P* 0.026).

#### Synthesis of the results on aerobic capacity

Three studies evaluated the effectiveness of aerobic capacity. In one study [21] (overall some concern RoB), aerobic capacity was evaluated with estimation of VO_2max_ (L/min) and found significant between-group difference after 6 weeks of exercise (MD, 0.16; 95%CI, 0.1 to 0.2, *P* < 0.0001) in favor of multimodal exercise intervention. In another study [23] (overall low RoB), aerobic capacity was evaluated using the peak oxygen consumption (VO_2PEAK_; ml/kg/min), showing a significant between-group difference after 12 weeks of exercise (MD, 5.2; 95%CI, 4.0 to 6.5, *P* < 0.001) in favor of supervised aerobic exercise. In the third study [26] (overall high RoB), aerobic capacity was evaluated using predicted peak oxygen consumption (VO_2peakpred_; ml/kg/min), finding no significant between-group difference after 10 weeks of exercise (MD, –3.9; 95%CI, –13.5 to 5.6, *P* 0.3490).

#### Synthesis of the results on health-related quality of life

Five studies evaluated the effectiveness of an exercise-based intervention on HRQoL, with one study [21] (overall some concern RoB) using two different questionnaires. Four studies [21, 24–26] (overall low RoB, overall high RoB) used the EORTC-QLQ-C30 (EORTC) to evaluate HRQoL and found no statistically significant improvements after 6-week supervised exercise intervention (MD, 2.2; 95%CI, –2.7 to 7.1, *P* = 0.400) [21], after a median duration of 21 days of exercise during hospitalization (50 vs 50, *P* = 0.660) [24], after 36 weeks of exercise (*P* = 0.113) [25] and after 10 weeks of exercise (MD, –13.2; 95%CI, –39.9 to 13.5, *P* = 0.2721) [26]. In contrast , one study [23] (overall low RoB) used the Functional Assessment of Cancer Therapy-Anemia (FACT-An) and found a significant between-group difference for overall QoL (MD, 9.5; 95%CI, 1.5 to 17.5, *P* = 0.021) after 12 weeks of aerobic exercise.

### Reporting biases

Reporting bias were found among the included studies. Two studies [25, 26] only presented some domains of the EORTC questionnaires without explaining the reasoning for reporting the chosen subscales. Likewise, without explanation, another study reported only a subset of the EORTC domains as baseline characteristics. One study [26] did not present data for the primary outcome (Vo2peak), and two studies did not present data for the secondary outcome (muscle strength) [22, 25]. A full description of the RoB judgments is presented in Figure 2.

#### Certainty of evidence

The certainty of evidence for all outcomes of the effectiveness of exercise-based interventions in adults with malignant lymphoma undergoing chemotherapy is found to be very low. The overall clinical heterogeneity of the studies, along with the risk of bias and variability in participants’ characteristics and treatment regimens, reduces confidence in the findings. Hence, based on the available evidence, caution is warranted in drawing definitive conclusions.

#### Ongoing trials

Ongoing studies are listed in Table 4.

**Table 4:** Ongoing RCT’s registered at ClinicalTrials.gov evaluation the effectiveness of exercise-based interventions on muscle mass, muscle strength, functional capacity, and health-related quality of life in adults with malignant lymphoma undergoing treatment.

Trial identifier	Study title	Country	Diagnose	Sample characteristics	Intervention	Primary outcome	Study completion
NCT 05595577	Improving Exercise Capacity with a Tailored Physical Activity Intervention (PALS)	USA	Non-Hodgkin Lymphoma and Hodgkin Lymphoma	Patients (no): 66 Age: 18 to 85 Years During treatment	1–2 sessions per week consisting of slow 15-min. aerobic warm-up, 20 min. of strength training, 15 min. of progressive intensity aerobic exercises and 10-min. cool down (stretching/toning) with elastic bands	Maximum oxygen uptake	2023 August
NCT 05556239	The Effect of Resistance Training in Patients with Malignant Lymphoma Undergoing Chemotherapy Treatment – the STAY STRONG TRIAL – a Randomized Controlled Trial.	Denmark	Non-Hodgkin Lymphoma and Hodgkin Lymphoma	Patients (no): 42 Age: +18 Years During treatment	Supervised resistance training program planned as 3 sessions per week of approximately 60 min. The resistance training program comprises 6 exercises for the major muscle groups, starting at 2 sets of 15 RM progressing to 4 sets of 8 RM	Lean body mass	2024 October
NCT 04670029	Impact of an Adapted Physical Activity Program on Event-free Survival in Patients with Diffuse Large-cell B Lymphoma Treated in 1^st^ Line	France	Diffuse large B-cell lymphoma	Patients (no): 186 Age: +65 Years During treatment	Supervised exercise program of 3 sessions per week. Comprising 2 sessions of 1 h muscle strengthening, stretching, flexibility and balance. And 1 aerobic exercise session of 1.5 h. Supplemented with unsupervised exercise sessions at home comprising one session of anaerobic exercise (elastic bands, free weights) and one session of aerobic exercise (Nordic walking) with declaration in a logbook	Event-free survival	2029 February

min: minute; no: number; RCT: randomized controlled trials; RM: repetitions maximum.

## Discussion

We conducted a systematic review and identified six RCTs that evaluated the effectiveness of an exercise-based intervention in patients diagnosed with NHL or HL undergoing chemotherapy. The body of evidence were characterized by extensive clinical heterogeneity in population, exercise interventions and methodological quality. Hence, the findings should be interpreted with caution. Nonetheless, exercise interventions were found to be feasible in patients diagnosed with malignant lymphomas undergoing treatment. There was no evidence suggesting elevated risks of adverse events due to exercise, although some incidents were reported, including one specific to the oncological context. While some studies suggest that exercising during chemotherapy may positively impact muscle mass, muscle strength, functional per-formance, aerobic capacity, and HRQoL outcomes, the certainty of the evidence is rated very low due to the high risk of bias. Initially, we aimed to focus solely on lymphoma patients undergoing chemotherapy. However, the systematic review includes a mixed population of malignant lymphomas (aggressive and indolent), other hematological diagnoses, and cancers. Consequently, due to the highly diverse medical treatments i.e. chemotherapy with or without stem cell transplantation, radiation, surgery, and immunotherapy, this systematic review includes various treatments. This diversity extends to patients not receiving chemotherapy. Unfortunately, none of the studies reported sub-group analyses specific to lymphomas. Although subgroup analysis would have been appropriate in all studies, attempts were made to acquire supplementary data specific to lymphoma patients by contacting all first and corresponding authors. However, only two authors replied, and only one provided relevant data. Consequently, we were not able to evaluate the effectiveness of exercise-based interventions specific to lymphoma patients. Maintaining adherence during cancer treatment can be challenging as patients often experience fatigue, poor well-being, and low energy levels. Three studies reported adherence rates of 65% to 92% indicating a positive impact, but these rates refer mainly to attendance, not whether the intervention met prescribed intensity and duration. Only one study reported the achieved intensity and duration as 90.7% and 99.0%, respectively [23]. In that specific study, strategies to improve adherence were incorporated with behavioral support techniques and practical arrangements such as flexible training hours, paid parking, and telephone follow-up after missed exercise sessions. It is unclear if these factors contributed to the high adherence. However, based on quantitative and qualitative findings in patients diagnosed with cancer undergoing treatment, these efforts are considered to positively influence adherence rate [29–31]. Three ongoing studies have the potential to enhance the evidence supporting the effectiveness of exercise intervention for lymphoma patients, with primary outcomes focusing on aerobic capacity, lean body mass and event-free survival. Notably, two of the studies focus exclusively on lymphoma patients, potentially enhancing population homogeneity while one study investigates a single mode supervised resistance intervention. Together, these studies may strengthen the evidence on the effectiveness of exercise interventions for lymphoma patients during treatment or confirm the consistency of earlier findings ultimately contributing to more robust recommendations for clinical practice.

### Strengths and limitations

Compared to similar systematic reviews [15, 32], the strengths of this study include the systematic approach, initiated by registering the review protocol at Prospero before study start. The review followed guidelines from the Cochrane Handbook [16] and reported in accordance with PRISMA guidelines [17]. Another strength is the rigorous search matrix, developed in close collaboration with an experienced health science librarian who conducted all electronic searches in the databases. Limitations include the inability to conduct subgroup analyses due to variations in the studies, which limits the ability to draw clear conclusions, as a meta-analysis was not feasible. Furthermore, a high proportion of the studies were judged to have a high RoB, primarily due to deviations from the intended intervention, missing outcome data, and selective reporting of outcomes. Therefore, caution is advised when interpretating the results. In addition, the review did not include feasibility studies, which may have provided more data on exercise adherence.

#### Implications for practice and implication for research

This review includes only six heterogeneous studies and provides no conclusion on the optimal exercise content, intensity, duration, or delivery. However, the evidence indicates that exercise is feasible, safe, and may have positive effects. More high-quality studies are needed to investigate the effectiveness of exercising on muscle mass, strength, functional performance, aerobic capacity, and HRQoL in these patients during treatment. Furthermore, subgroup analysis is highly recommended in future studies when including mixed hematological patient populations with different medical treatments. To assess intervention effectiveness, future studies should clearly describe the FITT factors (frequencies, intensities, time, and types) in accordance with reporting guidelines, while also ensuring and documenting intervention fidelity.

## Conclusion

Overall, exercise-based interventions during chemotherapy appear feasible and safe. However, given the very low quality of evidence, this systematic review cannot determine the effectiveness of exercise-based interventions on muscle mass, strength, functional capacity and performance, aerobic capacity or HRQoL in adults with malignant lymphomas.

## Supplementary Material

The effectiveness of exercise-based interventions on muscle mass, muscle strength, functional performance, aerobic capacity, and health-related quality of life in adults with malignant lymphoma undergoing chemotherapy: a systematic review of randomized controlled trials

## Data Availability

All data generated or analyzed during this study are included in this article and as supplementary files. Data can be made requested from the corresponding author.

## References

[1] Alaggio R, Amador C, Anagnostopoulos I, Attygalle AD, Araujo IBO, Berti E, et al. The 5th edition of the World Health Organization classification of haematolymphoid tumours: lymphoid neoplasms. Leukemia. 2022;36(7):1720–48. 10.1038/s41375-022-01620-2 35732829 PMC9214472

[2] Sung H, Ferlay J, Siegel RL, Laversanne M, Soerjomataram I, Jemal A, et al. Global cancer statistics 2020: GLOBOCAN estimates of incidence and mortality worldwide for 36 cancers in 185 countries. CA Cancer J Clin. 2021;71(3):209–49. 10.3322/caac.21660 33538338

[3] Pilleron S, Sarfati D, Janssen-Heijnen M, Vignat J, Ferlay J, Bray F, et al. Global cancer incidence in older adults, 2012 and 2035: a population-based study. Int J Cancer. 2019;144(1):49–58. 10.1002/ijc.31664 29978474

[4] Howlader N, Noone A, Krapcho M, Miller D, Brest A, Yu M, et al. SEER cancer statistics review. National Cancer Institute. Available from: https://seer.cancer.gov/csr/1975_2018/ based on November 2020 SEER data submission, posted to the SEER web site, April 2021. [Cited date: 12.03.2024]

[5] Vega MC, Laviano A, Pimentel GD. Sarcopenia and chemotherapy-mediated toxicity. Einstein. 2016;14(4):580–4. 10.1590/s1679-45082016md3740 28076611 PMC5221390

[6] Tanaka S, Imataki O, Kitaoka A, Fujioka S, Hanabusa E, Ohbayashi Y, et al. Clinical impact of sarcopenia and relevance of nutritional intake in patients before and after allogeneic hematopoietic stem cell transplantation. J Cancer Res Clin Oncol. 2017;143(6):1083–92. 10.1007/s00432-016-2336-8 28224299 PMC11819437

[7] Go SI, Park MJ, Song HN, Kim HG, Kang MH, Lee HR, et al. Prognostic impact of sarcopenia in patients with diffuse large B-cell lymphoma treated with rituximab plus cyclophosphamide, doxorubicin, vincristine, and prednisone. J Cachexia Sarcopenia Muscle. 2016;7(5):567–76. 10.1002/jcsm.12115 27104110 PMC4833756

[8] Nakamura N, Hara T, Shibata Y, Matsumoto T, Nakamura H, Ninomiya S, et al. Sarcopenia is an independent prognostic factor in male patients with diffuse large B-cell lymphoma. Ann Hematol. 2015;94(12):2043–53. 10.1007/s00277-015-2499-4 26385388

[9] Cruz-Jentoft AJ, Bahat G, Bauer J, Boirie Y, Bruyère O, Cederholm T, et al. Sarcopenia: revised European consensus on definition and diagnosis. Age Ageing. 2019;48(1):16–31. 10.1093/ageing/afy169 30312372 PMC6322506

[10] Anjanappa M, Corden M, Green A, Roberts D, Hoskin P, McWilliam A, et al. Sarcopenia in cancer: risking more than muscle loss. Tech Innov Patient Support Radiat Oncol. 2020;16:50–7. 10.1016/j.tipsro.2020.10.001 33385074 PMC7769854

[11] Pedersen BK, Saltin B. Exercise as medicine – evidence for prescribing exercise as therapy in 26 different chronic diseases. Scand J Med Sci Sports. 2015;25(Suppl. 3):1–72. 10.1111/sms.12581 26606383

[12] Campbell KL, Winters-Stone KM, Wiskemann J, May AM, Schwartz AL, Courneya KS, et al. Exercise guidelines for cancer survivors: consensus statement from international multidisciplinary roundtable. Med Sci Sports Exerc. 2019;51(11):2375–90. 10.1249/MSS.0000000000002116 31626055 PMC8576825

[13] Loughney LA, West MA, Kemp GJ, Grocott MP, Jack S. Exercise interventions for people undergoing multimodal cancer treatment that includes surgery. Cochrane Database Syst Rev. 2018;12(12):Cd012280. 10.1002/14651858.CD012280.pub2 30536366 PMC6517034

[14] Amatya B, Khan F, Lew TE, Dickinson M. Rehabilitation in patients with lymphoma: an overview of systematic reviews. J Rehabil Med. 2021;53(3):jrm00163. 10.2340/16501977-2810 33710351 PMC8814843

[15] AlJohi AA, Aljehani GH, AlSaeed SA, Alhoqail H, Mohammed J, Madi SM. Evidence-based exercises intervention in adults diagnosed with Lymphoma. Saudi Med J. 2022;43(5):441–50. 10.15537/smj.2022.43.5.20210894 35537731 PMC9280599

[16] Higgins J, Thomas J, Chandler J, Cumpston M, Li T, Page M, et al. Cochrane handbook for systematic reviews of interventions version 6.3 (updated February 2022). 2022. Available from: www.training.cochrane.org/handbook [Cited date: 21.07.2023] 10.1002/14651858.ED000142 PMC10284251 31643080

[17] Page MJ, Moher D, Bossuyt PM, Boutron I, Hoffmann TC, Mulrow CD, et al. PRISMA 2020 explanation and elaboration: updated guidance and exemplars for reporting systematic reviews. BMJ. 2021;372:n160. 10.1136/bmj.n160 33781993 PMC8005925

[18] Campbell M, McKenzie JE, Sowden A, Katikireddi SV, Brennan SE, Ellis S, et al. Synthesis without meta-analysis (SWiM) in systematic reviews: reporting guideline. BMJ. 2020;368:l6890. 10.1136/bmj.l6890 31948937 PMC7190266

[19] Sterne JAC, Savović J, Page MJ, Elbers RG, Blencowe NS, Boutron I, et al. RoB 2: a revised tool for assessing risk of bias in randomised trials. BMJ. 2019;366:l4898. 10.1136/bmj.l4898 31462531

[20] Altman DG, Bland JM. How to obtain the confidence interval from a P value. BMJ. 2011;343:d2090. 10.1136/bmj.d2090 21824904

[21] Adamsen L, Quist M, Andersen C, Møller T, Herrstedt J, Kronborg D, et al. Effect of a multimodal high intensity exercise intervention in cancer patients undergoing chemotherapy: randomised controlled trial. BMJ. 2009;339:b3410. 10.1136/bmj.b3410 19826172 PMC2762035

[22] Arrieta H, Astrugue C, Regueme S, Durrieu J, Maillard A, Rieger A, et al. Effects of a physical activity programme to prevent physical performance decline in onco-geriatric patients: a randomized multicentre trial. J Cachexia Sarcopenia Muscle. 2019;10(2):287–97. 10.1002/jcsm.12382 30829460 PMC6463460

[23] Courneya KS, Sellar CM, Stevinson C, McNeely ML, Peddle CJ, Friedenreich CM, et al. Randomized controlled trial of the effects of aerobic exercise on physical functioning and quality of life in lymphoma patients. J Clin Oncol. 2009;27(27):4605–12. 10.1200/JCO.2008.20.0634 19687337

[24] Oechsle K, Aslan Z, Suesse Y, Jensen W, Bokemeyer C, de Wit M. Multimodal exercise training during myeloablative chemotherapy: a prospective randomized pilot trial. Support Care Cancer. 2014;22(1):63–9. 10.1007/s00520-013-1927-z 23989498

[25] Streckmann F, Kneis S, Leifert JA, Baumann FT, Kleber M, Ihorst G, et al. Exercise program improves therapy-related side-effects and quality of life in lymphoma patients undergoing therapy. Ann Oncol. 2014;25(2):493–9. 10.1093/annonc/mdt568 24478323

[26] Munsie C, Ebert J, Joske D, Ackland T. A randomised controlled trial investigating the ability for supervised exercise to reduce treatment-related decline in adolescent and young adult cancer patients. Support Care Cancer. 2022;30(10):8159–71. 10.1007/s00520-022-07217-w 35792926 PMC9257117

[27] Buffart LM, Kalter J, Sweegers MG, Courneya KS, Newton RU, Aaronson NK, et al. Effects and moderators of exercise on quality of life and physical function in patients with cancer: an individual patient data meta-analysis of 34 RCTs. Cancer Treat Rev. 2017;52:91–104. 10.1016/j.ctrv.2016.11.010 28006694

[28] Guralnik JM, Simonsick EM, Ferrucci L, Glynn RJ, Berkman LF, Blazer DG, et al. A short physical performance battery assessing lower extremity function: association with self-reported disability and prediction of mortality and nursing home admission. J Gerontol. 1994;49(2):M85–94. 10.1093/geronj/49.2.M85 8126356

[29] Toohey K, Hunter M, Paterson C, Mortazavi R, Singh B. Exercise adherence in men with prostate cancer undergoing androgen deprivation therapy: a systematic review and meta-analysis. Cancers (Basel). 2022;14(10):2452. 10.3390/cancers14102452 35626058 PMC9139246

[30] Singh B, Spence R, Steele ML, Hayes S, Toohey K. Exercise for individuals with lung cancer: a systematic review and meta-analysis of adverse events, feasibility, and effectiveness. Semin Oncol Nurs. 2020;36(5):151076. 10.1016/j.soncn.2020.151076 33008682

[31] Purdy GM, Nanad R, Ternes L, Dolgoy ND, Sellar CM, Francis G, et al. Exercise preferences, barriers, and facilitators of individuals with cancer undergoing chemotherapy before stem cell transplantation: a mixed-methods study. Cancer Nurs. 2024;47(5):E287–97. 10.1097/NCC.0000000000001240 37058603

[32] Vermaete N, Wolter P, Verhoef G, Gosselink R. Physical activity, physical fitness and the effect of exercise training interventions in lymphoma patients: a systematic review. Ann Hematol. 2013;92(8):1007–21. 10.1007/s00277-013-1689-1 23408096

