# Supplementary material for: The effectiveness of exercise-based interventions on muscle mass, muscle strength, functional performance, aerobic capacity, and health-related quality of life in adults with malignant lymphoma undergoing chemotherapy: a systematic review of randomized controlled trials
Source: Acta Oncol. 2025 Jan 28;64:42056. doi: 10.2340/1651-226X.2025.42056 (PMC11808812; doi:10.2340/1651-226X.2025.42056)
Supplement: The effectiveness of exercise-based interventions on muscle mass, muscle strength, functional performance, aerobic capacity, and health-related quality of life in adults with malignant lymphoma undergoing chemotherapy: a systematic review of randomized controlled trials [file AO-64-42056-s1.pdf]

The effectiveness of exercise-based interventions on muscle mass, muscle strength, functional performance, aerobic capacity, and health-related quality of life in adults with malignant lymphoma undergoing chemotherapy: a systematic review of randomized controlled trials, Acta Oncologica, Charlotte Grønset, Magnus Nygaard Bech, Mary Jarden, Nina Høgdal, Martin Hutchings, Charlotte Suetta, Jan Christensen and e-mail of corresponding author: charlotte.groenset@regionh.dk

Supplementary material has been published as submitted. It has not been copyedited, or typeset by Acta Oncologica

Appendix A. The search strings for all databases and trials registers

Databases searched up to 15. Nov 2023
We ran a search on May 2022 and top-up searches in October 2022 and November 2023

Ovid MEDLINE(R) ALL

1 exp Lymphoma/
2 (lymphom* or nonhodgkin* or non-hodgkin* or hodgkin*).mp.
3 1 or 2
4 exp Exercise/
5 (exercise* or aerobic* or swim* or walk* or running or strength* train* or weight lift* or strength* program* or physical train* or rehabilitat* or weight train* or physical condition*).mp.
6 4 or 5
7 3 and 6
8 ((randomized controlled trial or controlled clinical trial).pt. or randomi?ed.ti,ab. or placebo.ab. or drug therapy.fs. or randomly.ab. or trial.ab. or groups.ab.) not (exp animals/ not humans.sh
9 7 and 8
Number of references 703

Embase Ovid

1 exp lymphoma/
2 exp Hodgkin disease/
3 (lymphom* or nonhodgkin* or non-hodgkin* or hodgkin*).mp.
4 1 or 2 or 3
5 exp exercise/
6 (exercise* or aerobic* or swim* or walk* or running or strength* train* or weight lift* or strength* program* or physical train* or rehabilitat* or weight train* or physical condition*).mp.
7 5 or 6
8 4 and 7
9 (randomized controlled trial/ or controlled clinical study/ or random$.ti,ab. or randomization/ or intermethod comparison/ or placebo.ti,ab. or (compare or compared or comparison).ti. or ((evaluated or evaluate or evaluating or assessed or assess) and (compare or compared or comparing or comparison)).ab. or (open adj label).ti,ab. or ((double or single or doubly or singly) adj (blind or blinded or blindly)).ti,ab. or double blind procedure/ or parallel

group$1.ti,ab. or (crossover or cross over).ti,ab. or ((assign$ or match or matched or allocation) adj5 (alternate or group$1 or intervention$1 or patient$1 or subject$1 or participant$1)).ti,ab. or (assigned or allocated).ti,ab. or (controlled adj7 (study or design or trial)).ti,ab. or (volunteer or volunteers).ti,ab. or human experiment/ or trial.ti.) not (((random$ adj sampl$ adj7 (cross section$ or questionnaire$1 or survey$ or database$1)).ti,ab. not (comparative study/ or controlled study/ or randomi?ed controlled.ti,ab. or randomly assigned.ti,ab.)) or (cross-sectional study/ not (randomized controlled trial/ or controlled clinical study/ or controlled study/ or randomi?ed controlled.ti,ab. or control group$1.ti,ab.)) or (((case adj control$) and random$) not randomi?ed controlled).ti,ab. or (Systematic review not (trial or study)).ti. or (nonrandom$ not random$).ti,ab. or Random field$.ti,ab. or (random cluster adj3 sampl$).ti,ab. or ((review.ab. and review.pt.) not trial.ti.) or (we searched.ab. and (review.ti. or review.pt.)) or update review.ab. or (databases adj4 searched).ab. or ((rat or rats or mouse or mice or swine or porcine or murine or sheep or lambs or pigs or piglets or rabbit or rabbits or cat or cats or dog or dogs or cattle or bovine or monkey or monkeys or trout or marmoset$1).ti. and animal experiment/) or (Animal experiment/ not (human experiment/ or human/)))
10 8 and 9
Number of references 734

Cinahl

S1 (MH "Lymphoma+")
S2 TX (lymphom* or nonhodgkin* or non-hodgkin* or hodgkin*)
S3 S1 OR S2
S4 (MH "Exercise+")
S5 (MH "Muscle Strengthening+")
S6 TX (exercise* or aerobic* or swim* or walk* or running or strength* train* or weight lift* or strength* program* or physical train* or rehabilitat* or weight train* or physical condition*)
S7 S4 OR S5 OR S6
S8 S3 AND S7
S9 (MH (randomized controlled trials) OR MH (double-blind studies) OR MH (single-blind studies) OR MH (random assignment) OR MH (pretest-posttest design) OR MH (cluster sample) OR TI (randomised OR randomized) OR AB (random*) OR TI (trial) OR (MH (sample size) AND AB (assigned OR allocated OR control)) OR MH (placebos) OR PT (randomized controlled trial) OR AB (control W5 group) OR MH (crossover design) OR MH (comparative studies) OR AB (cluster W3 RCT)) NOT ((MH (animals+) OR MH (animal studies) OR TI (animal model*)) NOT MH (human))
S10 S8 AND S9
Number of references 841

Cochrane Central Register of Controlled Trials

#1 MeSH descriptor: [Lymphoma] explode all trees
#2 nonhodgkin* or non-hodgkin* or hodgkin*
#3 lymphom*

The effectiveness of exercise-based interventions on muscle mass, muscle strength, functional performance, aerobic capacity, and health-related quality of life in adults with malignant lymphoma undergoing chemotherapy: a systematic review of randomized controlled trials, Acta Oncologica, Charlotte Grønset, Magnus Nygaard Bech, Mary Jarden, Nina Høgdal, Martin Hutchings, Charlotte Suetta, Jan Christensen and e-mail of corresponding author: charlotte.groenset@regionh.dk

#4	#1 or #2 or #3
#5	MeSH descriptor: [Exercise] explode all trees
#6	exercise*
#7	aerobic*
#8	swim*
#9	running
#10	strength* train*
#11	weight* lift*
#12	strength* program*
#13	physical train*
#14	rehabilitat*
#15	weight train*
#16	physical condition*
#17	#5 or #6 or #7 or #8 or #9 or #10 or #11 or #12 or #13 or #14 or #15 or #16
#18	#4 and #17, in trials

Number of references 425

ClinicalTrials.gov

exercise OR exercises OR training OR strengthening OR rehabilitation OR rehabilitate | Interventional Studies | OR hodgkins OR hodgkin OR non-hodgkins OR nonhodgkins OR non-hodgkin OR nonhodgkin

Applied Filters: Interventional

Number of references 168

WHO International Clinical Trials Registry Platform (ICTRP)

(exercise OR exercises OR training OR strengthening OR rehabilitation OR rehabilitate) AND (lymphoma OR lymphomas OR hodgkins OR hodgkin OR non-hodgkins OR nonhodgkins OR non-hodgkin OR nonhodgkin)

Number of references 92

Appendix B. Review authors' Risk of Bias judgements

In the study of Arrieta et al. high risk of bias was found based on the judgment of domain 3, "risk of bias due to missing outcome data". The decision is based on the reporting of both physical function (SPPS) and physical activity (IPAQ, METS). Regarding the primary outcome for physical function results for the 1 year follow up are only reported for 72 out of 151 participants randomized to the usual care group (UC) and for 74 out of 150 participants randomized to the intervention group (IG). The physical activity outcome was based on analysis of 80 out of 151 participants in the UC and 76 out of 150 participants in the IG. The description of this report is not explained sufficiently and therefore high risk of bias was judged according to the ROB2-guidelines.

In the study by Oechsle et al. high risk of bias was judged based on domain 2 and 3; "risk of bias due to deviation from the intended intervention" and "due to missing outcome data" for the patient reported outcome (the EORCT-QLQ-C30). No explanation of the statistical analysis for outcomes was presented leading to the use of per-protocol-analysis and the lack of description of missing outcome data from the randomized patients who dropped out during the study.

In the study by Streckmann et al. high risk of bias was judged on domain 2 and 5; "risk of bias due to deviations from the intended interventions" and "risk of bias in selection of the reported result". The study deviated from the exercise protocol which likely may have affected the outcome and due to lack of reasoning for only reporting selected subscales from the EORTC.

In the study by Munsie et al. high risk of bias was judged based on domain 5 "risk of bias in selection of the reported result". No analysis for the primary outcome and some domains for the EORTC is presented, which could lead to the fact that results are presented based on selected outcome reporting.

The study by Adamsen et al. overall risk of bias was assessed being of "some concern" based on the domain 4; "risk of bias in measurement of the objective outcomes" (muscle strength and cardiopulmonary fitness), since the outcome assessors were not blinded as they also performed the exercise sessions. This could lead to significant bias though we find it unlikely that the assessment

was influenced by knowledge of intervention received. For the subjective outcomes we also assessed “some concerns” since outcome assessors were aware of the received intervention.

The study from Courneya et al. was assessed being of overall “low” risk of bias for both objective (cardiopulmonary (Vo2)) and subjective outcomes (EORTC-QLQ-C30 (QoL)) for all domains.
